# Divisive gain modulation enables flexible and rapid entrainment in a neocortical microcircuit model

**DOI:** 10.1152/jn.00401.2019

**Published:** 2020-02-05

**Authors:** Christoforos A. Papasavvas, Andrew J. Trevelyan, Marcus Kaiser, Yujiang Wang

**Affiliations:** ^1^CNNP Lab, Interdisciplinary Computing and Complex BioSystems Group, School of Computing, Newcastle University, Newcastle upon Tyne, United Kingdom; ^2^Faculty of Medical Sciences, Newcastle University, Newcastle upon Tyne, United Kingdom; ^3^UCL Queen Square Institute of Neurology, Queen Square, London, United Kingdom; ^4^Department of Functional Neurosurgery, Ruijin Hospital, School of Medicine, Shanghai Jiao Tong University, Shanghai, China

**Keywords:** gain control, inhibitory interneurons, neocortical dynamics, neural entrainment, Wilson–Cowan model

## Abstract

Neocortical circuits exhibit a rich dynamic repertoire, and their ability to achieve entrainment (adjustment of their frequency to match the input frequency) is thought to support many cognitive functions and indicate functional flexibility. Although previous studies have explored the influence of various circuit properties on this phenomenon, the role of divisive gain modulation (or divisive inhibition) is unknown. This gain control mechanism is thought to be delivered mainly by the soma-targeting interneurons in neocortical microcircuits. In this study, we use a neural mass model of the neocortical microcircuit (extended Wilson–Cowan model) featuring both soma-targeting and dendrite-targeting interneuronal subpopulations to investigate the role of divisive gain modulation in entrainment. Our results demonstrate that the presence of divisive inhibition in the microcircuit, as delivered by the soma-targeting interneurons, enables its entrainment to a wider range of input frequencies. Divisive inhibition also promotes a faster entrainment, with the microcircuit needing less time to converge to the fully entrained state. We suggest that divisive inhibition, working alongside subtractive inhibition, allows for more adaptive oscillatory responses in neocortical circuits and, thus, supports healthy brain functioning.

**NEW & NOTEWORTHY** We introduce a computational neocortical microcircuit model that features two inhibitory neural populations, with one providing subtractive and the other divisive inhibition to the excitatory population. We demonstrate that divisive inhibition widens the range of input frequencies to which the microcircuit can become entrained and diminishes the time needed to reach full entrainment. We suggest that divisive inhibition enables more adaptive oscillatory activity, with important implications for both normal and pathological brain function.

## INTRODUCTION

Neural networks exhibit a diverse dynamic repertoire at macroscopic and microscopic scales (Buzsáki 2006; Wright and Liley 1996). Oscillatory activity is part of this repertoire and is understood to play a crucial role in information processing and many cognitive processes (Buzsáki 2006; Buzsáki and Draguhn 2004; Engel et al. 1991; Klimesch 1999; Traub et al. 1989; Vogels et al. 2005; Whittington et al. 2000). A crucial property of neural circuits is their ability to adjust their oscillation frequency to match a given stimulation frequency. This phenomenon is called neural entrainment. Entrainment to external rhythmic stimuli, such as sensory stimuli and transcranial magnetic stimulation, has been demonstrated through electroencephalography (EEG) and magnetoencephalography (MEG) recordings (Schwab et al. 2006; Tal et al. 2017; Thut et al. 2011). Neural entrainment indicates functional flexibility and is associated with healthy neural dynamics (Kösem et al. 2018; Riecke et al. 2018; Schwab et al. 2006; Spiegler et al. 2011; Zion Golumbic et al. 2013). Contrarily, impairment of entrainment is associated with abnormal brain conditions. For example, gamma-band entrainment is impaired in schizophrenia patients, and this impairment is a potential biomarker for the disorder (Brenner et al. 2003; Hamm et al. 2015; Krishnan et al. 2009).

Inhibition in cortical circuits shape neural dynamics and underlie oscillatory phenomena, such as entrainment (Traub et al. 1989; Vogels et al. 2005; Whittington et al. 2000). Generally, inhibitory mechanisms modulate the input-output functions of neurons (for a review see Silver 2010; Womelsdorf et al. 2014). Subtractive inhibition provides a hyperpolarizing effect and shifts the input-output functions to higher values of input, whereas divisive inhibition provides a divisive gain modulation that decreases the sensitivity (i.e., gain) in input changes (Ayaz and Chance 2009; Doiron et al. 2001; Litwin-Kumar et al. 2016). Experimental results from neocortical microcircuits linked these two inhibitory mechanisms with the dendrite-targeting (somatostatin-positive) and the soma-targeting (parvalbumin-positive) interneurons, respectively (Atallah et al. 2012; Pouille et al. 2009; Wilson et al. 2012). In addition, recent experimental and computational studies showed that it is precisely this difference in their target that enables their differential modulatory effects (Jadi et al. 2012; Pouille et al. 2013; Trevelyan and Watkinson 2005). Dendrite-targeting interneurons alone, interacting with local pyramidal cells, were recently shown to be sufficient for generating oscillatory activity in the primary visual cortex (Adesnik and Scanziani 2010; Hakim et al. 2018), corroborating the theoretical prediction that subtractive inhibition alone suffices to generate neural oscillations (Wilson and Cowan 1972). Meanwhile, divisive inhibition appears to increase the stability of oscillations in recurrent circuits (Chance and Abbott 2000; Papasavvas et al. 2015; Vida et al. 2006). However, it is still unknown whether divisive inhibition has any effect on neural entrainment.

Computational and theoretical studies on neural entrainment, which typically rely on neural mass models, have provided insights into the underlying mechanisms and testable predictions on the use of periodic stimulation (Herrmann et al. 2016; Masuda and Kori 2007; Roberts and Robinson 2012; Spiegler et al. 2011; Vierling-Claassen et al. 2008). For example, a model of entrainment in a single cortical area has been proposed as a framework to make predictions on photic driving experiments (Spiegler et al. 2011). Neural field theory has been applied on the corticothalamic system, aiming for a comprehensive interpretation of EEG responses to periodic visual stimulation in terms of the generating mechanisms (Roberts and Robinson 2012). Herrmann et al. (2016) have proposed through their simulations that periodic stimulation can be designed to manipulate endogenous oscillations in a predictable manner. Impaired entrainment, as seen in schizophrenia patients, has been modeled through GABA alterations for the investigation of the biophysical mechanisms involved (Vierling-Claassen et al. 2008). Furthermore, spike-timing-dependent plasticity with asymmetric learning windows has been shown to facilitate entrainment in a spiking network model (Masuda and Kori 2007). However, the role of divisive inhibition in neural entrainment has not been investigated.

Does divisive inhibition influence the entrainment of neocortical circuits? We will address this problem by using an extended version of the original Wilson–Cowan neural mass model of the neocortical microcircuit that incorporates gain control through divisive inhibition (Papasavvas et al. 2015). We hypothesize that divisive inhibition will improve the ability of a neocortical circuit to adjust its oscillating frequency to match an oscillating input. We will systematically vary the level of divisive inhibition in the model and report its impact on entrainment using two measures that capture how well, and how quickly, the microcircuit is entrained.

## MATERIALS AND METHODS

### 

#### Neocortical microcircuit model.

**Fig. 1.**
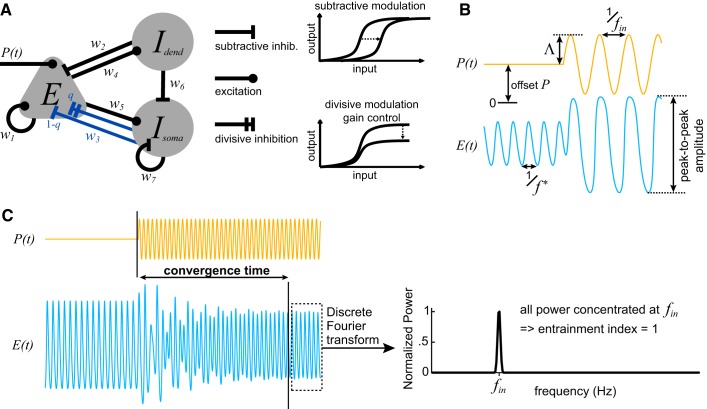
Neocortical microcircuit model and entrainment measures. *A*: schematic of the neocortical circuit model. It features one excitatory (*E*) and two inhibitory subpopulations, one dendrite-targeting (*I*_dend_) and one soma-targeting interneuronal population (*I*_soma_), and the weights (*w*) of their connections. Notice that the nature of inhibition delivered from *I*_soma_ to *E* is governed by the divisiveness parameter 0 ≤ *q* ≤ 1 (shown in blue). It ranges from completely subtractive (*q* = 0) to completely divisive (*q* = 1) modulation. Schematics of these two types of input-output modulation are also shown. *B*: example of entrainment. Initially, the circuit receives a constant input, *P*(*t*) = *P*, and oscillates at its natural frequency, *f*^∗^ [represented by the activity of the excitatory population *E*(*t*)]. The input becomes a sinusoidal wave with amplitude Λ and input frequency *f*_in_. The microcircuit is successfully entrained to the input frequency. *C*: entrainment of the microcircuit can take several oscillation cycles, as shown in this example. Convergence time is calculated as the time needed for the activity to converge to the final limit cycle. The entrainment index is then calculated from the power spectrum of the signal *E*(*t*) after its convergence.

The model used in this study is an extended version of the spatially localized Wilson–Cowan model (Wilson and Cowan 1972). This model of the neocortical microcircuit features one excitatory population, *E*, and two inhibitory subpopulations: one dendrite-targeting, *I*_dend_, and one soma-targeting interneuronal population, *I*_soma_ (see Fig. 1*A*). The two inhibitory subpopulations differ in the type of inhibitory modulation that they provide to the excitatory population. The *I*_dend_ always delivers subtractive inhibition, that is, subtractive modulation of the input-output function of its target: shifting the curve to higher input values. The *I*_soma_ can deliver a combination of subtractive and divisive inhibition, that is, divisive gain modulation, onto the excitatory population: decreasing the slope and maximal output of the function (see modulation schematics in Fig. 1*A*).

The original Wilson–Cowan model features the subtractive modulation but, to introduce the divisive modulation into the model, we need to define a sigmoidal input-output function that accepts three variables: the input or drive, *x*; the subtractive modulation, Θ; and the divisive modulation, A:

(1) $$F_{e,i}\left(x,\text{Θ},\text{Α}\right)=\left(\frac{\alpha_{e,i}}{\alpha_{e,i}+q\text{Α}}\right)\left[\frac{1}{1+\text{exp}\left\{-\alpha_{e,i}\left[x-\left(\theta_{e,i}+\text{Θ}+\left(1-q\right)\text{Α}\right)\right]\right\}}-\frac{1}{1+\text{exp}\left(\alpha_{e,i}\theta_{e,i}\right)}\right],$$

where *e* stands for excitatory and *i* stands for inhibitory. The function is defined with all inputs being real non-negative values. The constant θ*_e,i_* is the minimum displacement of the input-output function on the *x*-axis for the respective population, representing the default state when there is no subtractive inhibition that displaces the function further. The constant α*_e,i_* is the maximum gain of the sigmoidal function for the respective population, representing the default state when there is no divisive inhibition that decreases the function’s gain. Notice how the divisiveness parameter *q* ∈ [0, 1] governs the effect of the divisive modulation A. Only when *q* = 1 is the modulation purely divisive. A lower value leads to a combination of subtractive and divisive modulation, and it becomes purely subtractive with *q* = 0. In addition, notice that when A = 0, this function is equivalent to the sigmoidal function used in the original Wilson–Cowan model (Wilson and Cowan 1972). The input-output function defined here is a variation of the function used in Papasavvas et al. (2015), and we explained its exact derivation in Papasavvas and Wang (2019).

By using the input-output function *F_e,i_*, the neocortical microcircuit is described by the following system of ordinary differential equations:

$$\tau \frac{dE\left(t\right)}{dt}=-E\left(t\right)+\left(k_{e}-E\left(t\right)\right)F_{e}\left(w_{1}E\left(t\right)+P\left(t\right),w_{2}I_{dend}\left(t\right),w_{3}I_{soma}\left(t\right)\right),$$

$$\tau \frac{dI_{dend}\left(t\right)}{dt}=-I_{dend}\left(t\right)+\left(k_{i}-I_{dend}\left(t\right)\right)F_{i}\left(w_{4}E\left(t\right),0,0\right),$$

(2) $$\tau \frac{dI_{soma}\left(t\right)}{dt}=-I_{soma}\left(t\right)+\left(k_{i}-I_{soma}\left(t\right)\right)F_{i}\left(w_{5}E\left(t\right),w_{6}I_{dend}\left(t\right)+w_{7}I_{soma}\left(t\right),0\right),$$

where, analogous to the original Wilson–Cowan model,

(3) $$k_{e,i}=\underset{x\to \infty }{\lim}F_{e,i}\left(x,0,0\right)=\frac{\exp\left(\alpha_{e,i}\theta_{e,i}\right)}{1+\exp\left(\alpha_{e,i}\theta_{e,i}\right)}.$$

Figure 1*A* shows a schematic for the neocortical microcircuit indicating excitatory and inhibitory connections and their weights, *w*. To constrain the parameters of this model, we used the findings in the primary visual cortex by Pfeffer et al. (2013). First, *I*_dend_ inhibits *I*_soma_, but not reciprocally. Second, there is self-inhibition for *I*_soma_ but not for *I*_dend_. Third, some weight values are set relative to *w*_3_: *w*_7_ = *w*_3_, *w*_2_ = 0.54*w*_3_, and *w*_6_ = 0.33*w*_3_.

The parameters used for the input-output functions *F_e,i_* (*Eq. 1*) are θ*_e_* = 4, θ*_i_* = 3.7, α*_e_* = 1.3, and α*_i_* = 2. These are the values used in the original Wilson–Cowan model (Wilson and Cowan 1972). The time constant was set to τ = 0.05. This value was chosen based on the response of the neocortical microcircuit model to an instantaneous input (see Supplemental Fig. S1; see https://doi.org/dkwh), which has a characteristic half-life *t*_1_*_/_*_2_ = 39.8 ms, thus approximating the response of electrically stimulated neocortical microcircuits in electrophysiological experiments (Alfonsa et al. 2015).

The microcircuit model was specifically designed to investigate the influence of divisive inhibition, as delivered by the soma-targeting inhibitory population *I*_soma_, on the entrainment of the microcircuit. The divisiveness parameter *q* is incorporated into the model to create a continuum between the two extremes: from purely subtractive inhibition to purely divisive inhibition delivered by *I*_soma_. By varying parameter *q* from 0 to 1, we can investigate quantitatively, by the use of appropriate measures (see below), how entrainment is influenced by a systematic increase of the divisiveness of inhibitory modulation. Note that the divisiveness parameter governs only the modulation delivered by *I*_soma_ onto *E*, while *I*_dend_ always delivers purely subtractive modulation, and this is because divisive gain modulation has been linked with the soma-targeting interneurons in neocortex (Atallah et al. (2012); Pouille et al. (2009); Wilson et al. (2012); however, see also Seybold et al. (2015)). Despite the fact that divisiveness is exclusively delivered by *I*_soma_, we still incorporate *I*_dend_ in the model to allow comparison with previous modeling results.

Neural mass models can operate in many different regimes, even during a constant input. Systematic analysis of their dynamics showed that they can be in a (robust) stable state, either a stable fixed point or a stable limit cycle (Grimbert and Faugeras 2006; Spiegler et al. 2010; Wilson and Cowan 1972). They can also be in a marginally stable fixed point, that is, a critical point between an absorbing state and an activated state that reflects the evidence of criticality in experimental data (Cowan et al. 2016). Even chaotic regimes can be achieved in a neural mass model with at least three variables (Papasavvas et al. 2015; Spiegler et al. 2011). However, in this study, as in previous work (Spiegler et al. 2011), we investigate the phenomenon of entrainment only in the regime of a stable limit cycle, that is, with an existing oscillation before any oscillatory drive. This is because the effect of entrainment, in a biological sense, is usually considered as an input-driven adaptation of an already existing oscillation.

#### Entrainment measures.

**Fig. 2.**
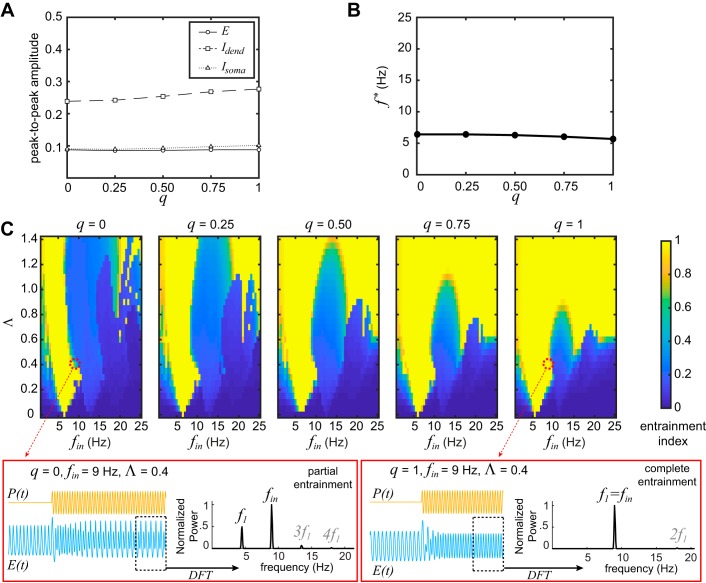
Effect of parameter *q* on the entrainment index. *A*: peak-to-peak amplitude for all 3 populations while the input is constant using the example parameter set. The peak-to-peak amplitudes of all populations change very little with varying *q*. *B*: natural frequency, f^*^, of the microcircuit while the input is constant. This frequency does not change substantially with increasing *q*. *C*: heat maps of entrainment index for different combinations of the oscillatory input frequency, *f*_in_, and amplitude, Λ, and for different values of *q*. The areas of complete entrainment (index = 1) get wider as *q* increases (higher divisiveness). *Insets* (red markers) show examples of a partial entrainment (multipeak spectrum) and a complete entrainment (single-peak spectrum). The peaks marked 2*f*_1_, 3*f*_1_, and 4*f*_1_ (shown in gray) are removed before the calculation of the entrainment index (see materials and methods). Note that all model parameters and the input settings are identical except for the value of *q*. We used the example parameter set for all panels. DFT, discrete Fourier transform; *E*, excitatory population; *E*(*t*), activity of excitatory population; *I*_dend_, dendrite-targeting inhibitory subpopulation; *I*_soma_, soma-targeting inhibitory subpopulation; *P*(*t*), input.

To measure the entrainment of our microcircuit model to an oscillatory input with frequency *f*_in_, we calculate the discrete Fourier transform (DFT, using fft in MATLAB) of the response of the excitatory population *E*(*t*) after its convergence to the long-term behavior. The discrete Fourier transform is used to estimate the power spectral density (PSD) of the signal. All the PSD peaks are found, and then we determine their corresponding frequency and power values. The PSD peaks are sorted based on their power, and we check for each peak whether its corresponding frequency is a multiple of another peak with higher power. In this case, the peak with the lower power is considered to be a harmonic peak of the high-power frequency and it is removed, unless it corresponds to the input frequency *f*_in_. The input frequency is always preserved in the PSD so that any subharmonic entrainment can be detected. After the harmonic peaks are removed, the power among all the remaining peaks is normalized so that it sums up to 1. This normalization gives the proportion of power concentrated at each peak. The entrainment index is defined as the proportion of power that is concentrated at *f*_in_. This measure can take values from 0 to 1, with 0 denoting no entrainment and 1 denoting complete entrainment, while values in between indicate partial entrainment. Since the discrete Fourier transform always produces spectral peaks at higher harmonics when the signal is not a perfect sinusoid, the removal of such harmonics before normalization ensures that the entrainment index is not influenced by how much the output of the system resembles a perfect sinusoid. Figures 1*C* and 2*C* provide examples of the entrainment index calculation. In Fig. 2*C*, the peaks marked as 2*f*_1_, 3*f*_1_, and 4*f*_1_ are removed before power normalization and the calculation of the entrainment index.

**Fig. 3.**
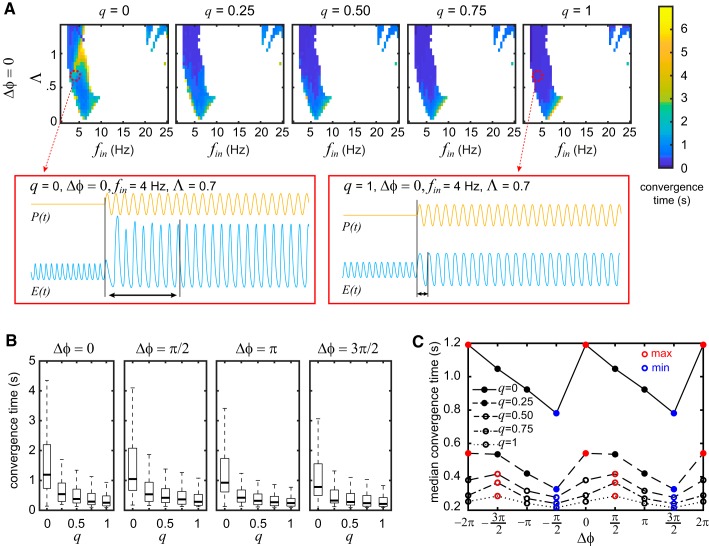
Effect of parameter *q* and the initial phase difference ∆φ on the convergence time. *A*: heat maps of convergence time for the same example parameter set as in Fig. 2. This panel shows only the heat maps produced with ∆φ = 0. These heat maps include only the combinations of input frequency, *f*_in_, and amplitude, Λ, that produce complete entrainment (entrainment index *>* 0.98) for all values of *q*, as shown in Fig. 2. Increasing the parameter *q* leads to faster entrainment in most cases. *Insets* (red markers) show examples of a slow and a fast convergence to the fully entrained state. Note that the model parameters and the input settings are the same except for the value of *q*. *B*: boxplots summarizing the distribution of the convergence time values for different ∆φ values. Median convergence time (thick line inside box) decreases with increasing values of *q*. Outliers are omitted in these boxplots to aid visual clarity. *C*: fluctuations of median convergence time as ∆φ changes. Its minimal (min) and maximal (max) values for each value of *q* are indicated with blue and red markers, respectively. Notice that the fastest convergence occurs with ∆φ = 3π/2 for all values of *q*, whereas the slowest convergence occurs at ∆φ = 0 for low values of *q* and at ∆φ = π/2 for high values of *q*. *E*(*t*), activity of excitatory population; *P*(*t*), input.

The convergence time is a measure of entrainment that quantifies the ability of the microcircuit to rapidly adapt to the oscillating input. It is only used to additionally characterize a complete entrainment (entrainment index *>* 0.98) by a measure of speed. Here we define the convergence time as the time needed for the population activity (considering all 3 state variables) to converge to its long-term behavior (limit cycle). To measure the convergence time, we first define the limit cycle as the set of unique points in phase space that describe the activity during the last 3 s of the 20-s-long simulation (by using uniquetol in MATLAB with tolerance 0.001). By scanning backward through the system’s activity, we then find the first time point when there is a system’s state that deviates from the limit cycle (by using ismembertol in MATLAB with tolerance 0.01). Examples of such measurements of convergence time are illustrated in Figs. 1*C* and 3*A*.

#### Sampling the parameter space and finding local optima.

To investigate the effect of the divisiveness parameter *q* on the entertainment measures, we had to set the parameters of the microcircuit such that its intrinsic oscillatory activity [response to constant input *P*(*t*) = *P*] at *q* = 0 and *q* = 1 is comparable. We developed a strategy to sample the parameter space for the parameters {*P*, *w*_1_, *w*_3_, *w*_4_, *w*_5_} and to produce a collection of valid parameter sets. The other connectivity parameters {*w*_2_, *w*_6_, *w*_7_} are set relative to *w*_3_ (see above). For a parameter set to be considered valid for investigation, it must comply with the following conditions:

1. It follows the connectivity relationships derived from Pfeffer et al. (2013).
2. It produces oscillatory behavior for both *q* = 0 and *q* = 1 with constant input, *P*(*t*) = *P*.
3. The difference between peak-to-peak amplitude (see Fig. 1) of *E*(*t*) at *q* = 0 and *q* = 1 is less than 0.001.
4. The peak-to-peak amplitude of *I*_soma_(*t*) is at least as large as the peak-to-peak amplitude of *E*(*t*) at both *q* = 0 and *q* = 1.

Note that it is necessary to apply a limitation on the amplitude of *I*_soma_(*t*) (*condition 4*) because it is possible to have connectivity parameters that diminish the amplitude of *I*_soma_(*t*) while maintaining oscillations in the other two populations. In this case, the system is reduced to the classic two-dimensional *E*–*I* system (Wilson and Cowan 1972). A similar limitation on the *I*_dend_(*t*) is not necessary because sustained oscillations in the microcircuit are not possible with a diminished activity in *I*_dend_(*t*) at *q* = 1. To produce a collection of such parameter sets, we randomly sampled a hypercube in the parameter space defined by the intervals: *P* ∈ [0, 5] and *w_k_* ∈ [0, 35] for *k* ∈ {1, 3, 4, 5}. After we generated the rest of the parameters based on *condition 1*, the system was simulated, and only those samples that complied with *condition 2* were saved. These sets were then used as the starting points for an optimization problem. The optimization problem was to minimize the difference between the peak-to-peak amplitudes of *E*(*t*) at *q* = 0 and *q* = 1 (*condition 3*) while maintaining the peak-to-peak amplitude of *I*_soma_(*t*) at least as large as the peak-to-peak amplitude of *E*(*t*) (*condition 4*). A penalty term was used during optimization to guarantee that *condition 4* is met and that the parameters do not deviate from the hypercube defined above. The optimization problem was solved using the nonlinear programming solver fminsearch in MATLAB.

A valid parameter set was initially produced through this method and used as an example set for the results in Figs. 2 and 3. The example set is {*P =* 1.428, *w*_1_ = 24.368, *w*_3_ = 9.677, *w*_4_ = 27.249, *w*_5_ = 30.913}. The other connectivity parameters are set relative to *w*_3_, that is, *w*_2_ = 5.225, *w*_6_ = 3.193, and *w*_7_ = 9.677.

For the purpose of generalizing the findings across different regions of the parameter space, we produced a collection of valid sets after an extensive sampling. The random sampling of 250,000 parameter sets generated a collection of 1,227 parameter sets that comply with *conditions 1* and *2*. From those sets, 981 were considered unique with tolerance 0.05 (using uniquetol in MATLAB). These unique sets were then used as the starting points for the optimization problem. From the 981 unique starting points, 359 converged to a parameter set that complied with all four conditions, and 138 of them were considered unique with tolerance 0.05.

#### Simulation protocol.

We simulated the following scenario: the microcircuit receives a constant excitatory input *P*(*t*) = *P* for at least 10 s, and then the input becomes sinusoidal *P*(*t*) = *P* + Λsin(2π*f*_in_*t*) (schematic in Fig. 1*B*). During the first 10 s, the microcircuit converges to its initial limit cycle, oscillating at its intrinsic (or natural) frequency *f*^∗^. The input changes from constant to sinusoidal at the first instance after the tenth second such that the initial phase difference between ongoing oscillation and sinusoidal input is to a specified value (∆φ = {0, π/2, π, 3π/2}).

#### Implementation and code availability.

The simulations and analyses were carried out in MATLAB version R2017b (The MathWorks, Inc., Natick, MA) and the bifurcation diagrams were produced using MATCONT 6.11 (Dhooge et al. 2008). The model and simulation code can be found in https://github.com/cpapasavvas/Entrainment2019.

## RESULTS

To test how divisive inhibition affects the entrainment of neocortical microcircuits, we ran simulations during which the constant input to the model becomes sinusoidal and analyzed the response of the model to this change (see materials and methods, *Simulation protocol*). More specifically, we explored how the divisiveness parameter *q* affects the ability of the microcircuit to become entrained to different oscillatory inputs (different amplitude, Λ, and frequency, *f*_in_, values) using a metric we term the “entrainment index.” We further measured the speed at which entrainment happens, which we term the “convergence time” (see materials and methods, *Entrainment measures*).

### 

#### Effect of divisiveness q on the entrainment index.

To ensure that the model displays comparable behavior between *q* = 0 (fully subtractive inhibition) and *q* = 1 (fully divisive inhibition), we show the peak-to-peak amplitudes of the limit cycle for all three state variables at different *q* values in Fig. 2*A*. The peak-to-peak amplitude barely changes for either *E* or *I*_soma_, while there is a small increase for *I*_dend_ with increasing value of *q*. The natural frequency *f*^∗^ of the microcircuit is also minimally influenced, ranging from ~6.5 to 5.5 Hz with an increasing value of *q*, as shown in Fig. 2*B*. Therefore, increasing *q* from 0 to 1 barely changes the characteristics of the intrinsic oscillations in this example parameter setting.

Using this parameter setting, we then tested how entrainment varies at different levels of *q*. Figure 2*C* suggests that increased *q* (higher divisiveness) dramatically enhances the microcircuit’s entrainment to an oscillatory input. The heat maps of entrainment index show that, for low input amplitude values (Λ *<* 0.2), the microcircuit becomes entrained to input frequencies that are close to its natural frequency *f*^∗^, but entrainment fails completely with input frequencies that deviate more than 3 Hz from *f*^∗^. This is true for any value of *q*. However, for higher values of Λ, the range of input frequencies that can entrain the microcircuit becomes wider as the value of *q* increases. The ellipsoid area shrinking with increasing *q* in the middle of the heat map represents the regime of subharmonic entrainment 1:2 (Herrmann et al. 2016; Roberts and Robinson 2012). In this regime, which is also evident in experimental data (Herrmann 2001), the microcircuit oscillates at the subharmonic frequency *f*_in_*/*2 (i.e., close to its natural frequency *f*^∗^). Notice that increasing *q* shrinks this regime but does not diminish it completely. The *insets* in Fig. 2*C* show an example of how a partial entrainment at *q* = 0, indicated by a multipeak spectrum, can become a complete entrainment at *q* = 1, indicated by a single-peak spectrum, just by increasing the divisiveness parameter *q*.

Note that the results reported here are produced with an initial phase difference between the input oscillation and the intrinsic oscillation of ∆φ = 0. There is no substantial effect of ∆φ on the entrainment index, especially for high *q* values. Minor differences in the heat maps are found for low *q* values (see Supplemental Fig. S3; see https://doi.org/dkwj).

We also repeated the same analysis as above for the relative change in oscillation power (as opposed to entrainment index; see Supplemental Fig. S2; https://doi.org/dkwk). At least for this example set of parameters, the relative change in power reaches much higher values for *q* = 0 compared with higher *q* values. This could be due to its qualitatively different bifurcation diagram, also shown in Supplemental Fig. S2 (see https://doi.org/dkwk).

#### Effect of divisiveness q on the convergence time.

After establishing the regions of input parameter space for which entrainment occurs, we next investigated how quickly this happens using the measure of convergence time. With the same example model parameters as before, we produced heat maps showing the convergence time for different combinations of Λ, *f*_in_, and *q* (see Fig. 3*A*). Note that these heat maps include only those regions of the Λ *× f_i_* space that lead to a complete or almost complete entrainment with index *>* 0.98 for all values of *q*, as reported in Fig. 2*C*. In other words, these results focus on how fast the microcircuit converges to the fully entrained state for different values of *q*. Note that such high values of entrainment index always represent 1:1 entrainment. From the heat maps, it is evident that increasing the value of *q* (higher divisiveness) decreases the convergence time. An example of such speeding up of entrainment is shown in the *insets* of Fig. 3*A*, where the only parameter that changes is the value of *q*.

The distributions of the convergence time for different initial phase differences (∆φ) are also shown in the boxplots of Fig. 3*B*. Notice that the median value (bold line in box) of the convergence time steadily decreased as *q* increased for all values of ∆φ.

The value of *q* has an additional effect on the convergence time regarding the initial phase difference ∆φ. As shown in Fig. 3*C*, the median convergence time for each *q* fluctuates depending on the value of ∆φ. The lowest median convergence time is recorded at ∆φ = 3π/2 for all values of *q*. However, the highest median convergence time depends on *q*. For low values of *q*, the slowest entrainment is found at ∆φ = 0, whereas the slowest entrainment for high values of *q* is found at ∆φ = π/2.

We also investigated the final phase difference between input and population activity after the convergence to a fully entrained state. The main purpose was to explore whether there is any anti-phase entrainment (final phase difference close to π; see also Borisyuk et al. 1995) and whether *q* influences its appearance. The results suggest that there is no instance of anti-phase entrainment, at least for this example parameter set (see Supplemental Fig. S4; see https://doi.org/dkwm).

#### Generalization of the findings to other parameter sets.

**Fig. 4.**
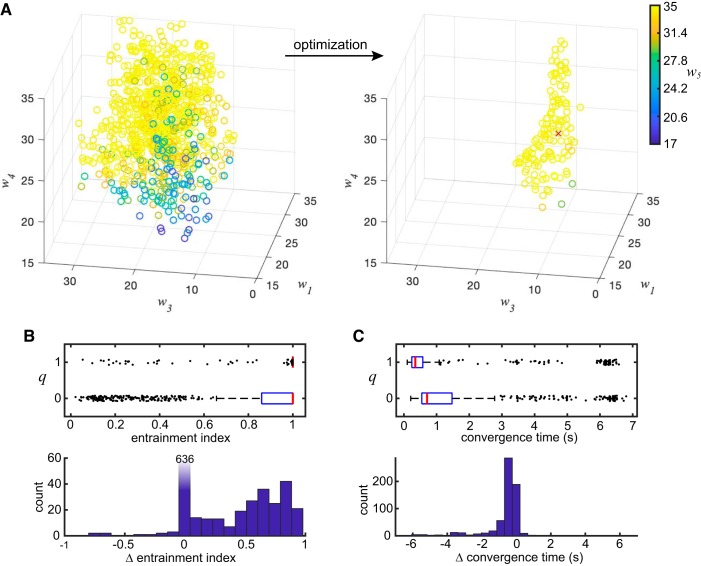
Generalizing the findings across the parameter space. *A*: visualization of the random sampling of the parameter space before the optimization (*left*) and the resulting local optima (*right*). Only 4 of 5 parameters are visualized for simplicity. Distributions of parameter *P* (not shown): 1.853 ± 0.862 before optimization, 1.230 ± 0.354 after optimization (mean ± SD). Red cross indicates the parameter set used for the results shown in Figs. 2 and 3. *B*: distribution of entrainment indices for *q* = 0 and *q* = 1 (*top*). Despite the fact that the median value (red line) is 1 in both cases, almost the whole distribution is concentrated at 1 for *q* = 1, but not for *q* = 0, as indicated by the interquartile ranges (box). The paired differences (between *q* = 1 and *q* = 0; *bottom*) indicate that the entrainment index generally increases with *q* for the parameter sets tested. *C*: distribution of convergence times for *q* = 0 and *q* = 1 (*top*). The paired differences (*bottom*) indicate that the entrainment generally happens faster while the divisiveness parameter *q* is increased across the sampled parameter space.

Moving beyond the example parameter set, to generalize the findings in the parameter space, we followed the approach described in materials and methods. Briefly, a collection of valid parameter sets was produced, and we tested whether the findings hold across this collection. The scatter plots in Fig. 4*A* represent a four-dimensional visualization of the collection of the unique parameter points before and after the optimization (see materials and methods for details). The red cross indicates the parameter set used for the results reported above.

The optimization procedure yielded 138 unique parameter sets that fulfilled the criteria of generating comparable intrinsic oscillations between *q* = 0 and *q* = 1 (see materials and methods). For each one of these 138 unique parameter sets, we measured the natural frequencies of the microcircuit for *q* = 0 and *q* = 1 when there is a constant input *P*(*t*) = *P*, that is, $f_{q=0}^{*}$ and $f_{q=1}^{*}$. The distribution of these intrinsic frequencies is limited to δ and θ band frequencies (see Supplemental Fig. S5; see https://doi.org/dkwn). By using these values and the parameter *P*, we generated a set of nine input settings, defined by the *combination* $\Lambda =\left\{0.1P,0.3P,0.5P\right\}\;\times f_{in}=\{f_{q=0}^{*},(f_{q=0}^{*}+f_{q=1}^{*})/2,f_{q=1}^{*}\}$. Note also that the results in this subsection were produced with ∆φ = 3*π/*2. As the results in Fig. 3C suggest, this value is an unbiased value because it promotes a faster convergence for all values of *q*.

From the 138 unique parameter sets, some produced oscillations after the onset of the oscillating input for all nine different input settings, while others produced oscillations only for low-amplitude settings (Λ = 0.1*P*). Only a subset of 96 parameter sets that produced oscillations for both *q* = 0 and *q* = 1 using all nine different input settings was used for the generalization results. This means that we produced 96 *×* 9 = 864 pairs of entrainment indices, one for *q* = 0 and another for *q* = 1. The distribution of the entrainment indices (*top*) and their paired differences (*bottom*) are shown in Fig. 4*B*. For both *q* = 0 and *q* = 1, the median entrainment index is 1 (red lines), but the interquartile range (box) starts and finishes at 1 for *q* = 1, whereas it ranges from 0.85 to 1 for *q* = 0. From the paired differences in Fig. 4*B*, *bottom*, it is evident that the increase of *q* (higher divisiveness) leads to a higher index and thus enhanced entrainment in general, at least for the sampled parameter space.

From the 864 combinations of model parameters and input settings that were used to produce the results in Fig. 4*B*, the great majority (618 combinations) led to a complete or almost complete entrainment (index > 0.98) for both *q* = 0 and *q* = 1. This subset was then used to explore whether the value of *q* has an impact on the time needed to converge to the fully entrained state (as in Fig. 3*A*). Figure 4*C*, *top*, shows that the convergence time for *q* = 1 (fully divisive) tends to be substantially lower than the convergence time for *q* = 0 (fully subtractive). Figure 4*C*, *bottom*, shows the distribution of paired differences: convergence time at *q* = 1 minus convergence time at *q* = 0. Their paired differences show that this finding, of models with divisive inhibition (*q* = 1) converging faster than those with subtractive inhibition (*q* = 0), occurs over a wide range of model parameters and so should be regarded as a general feature in this type of models.

## DISCUSSION

Our findings demonstrate that divisive inhibition enhances the entrainment of neocortical microcircuits, where a faster convergence and a wider range of entrainment frequencies are enabled. Indeed, the circuit becomes more flexible as the divisiveness parameter *q* increases.

These findings were generalized by sampling the parameter space and comparing the entrainment measures between the two extremes: *q* = 0 (fully subtractive) and *q* = 1 (fully divisive). The fact that the effect of the divisiveness parameter was observed across a wide range of parameter settings suggests the robustness of the effect. In addition, the results in Figs. 2 and 3, which include intermediate values of *q*, suggest that this effect is monotonic and even intermediate values, such as *q* = 0.5, can still facilitate entrainment. These intermediate values may represent the real biological system better, where a mixture of subtractive and divisive modulation is observed, as delivered by either soma-targeting or dendrite-targeting interneurons (Seybold et al. 2015).

It is important to clarify that the divisiveness parameter *q* governs only the inhibition delivered from one of the inhibitory subpopulations. Even when this inhibition is fully divisive (*q* = 1), there is still subtractive inhibition delivered to the excitatory population by the other inhibitory subpopulation. The former subpopulation represents the parvalbumin-expressing interneurons, whereas the latter represents the somatostatin-expressing interneurons, based on the experimental evidence (Atallah et al. 2012; Pouille et al. 2009; Wilson et al. 2012). The fact that both modulations are active may underlie the production of flexible dynamics in the microcircuit (see also, combining subtractive and divisive negative feedback processes; Huguet et al. 2017). Note that subtractive and divisive modulation, as modeled here, are “orthogonal” to each other. Subtractive inhibition modulates the position of the input-output curve along the input axis without influencing its shape, while divisive inhibition modulates its slope and maximal output without introducing any displacement. We hypothesize that it is exactly this orthogonality that allows for more flexibility in entrainment. Future work will need to investigate this hypothesis: simultaneous subtractive and divisive modulations are necessary for oscillatory flexibility.

Our model, as every neural mass model, is an abstract and simplified description of the real biological system, and it is unknown whether the findings translate well to the physical neocortical circuits or even to alternative models, e.g., one with spiking dynamics. A follow-up study could investigate whether this effect holds in a spiking neural network that exhibits divisive gain modulation. For this investigation, a network composed of leaky integrate-and-fire neurons, as in Ly and Doiron (2009), could be used. Ly and Doiron do not include any explicit models of dendritic/subtractive or somatic/divisive inhibition; however, the network exhibits divisive gain modulation as a result of balanced conductance fluctuations (Ly and Doiron 2009). Alternatively, we could use a biophysically detailed model with multicompartmental neurons, explicitly modeling inhibition at the soma and the dendrites as in Jadi et al. (2012) and Pouille et al. (2013). In both studies, subtractive and divisive gain modulation arise as a result of the relative location between the excitatory and inhibitory conductances. The different placement of inhibitory conductances, relative to the location of the excitatory drive, was shown to have different modulatory effects on the input-output function of the neuron due to the different changes on the electrotonic properties of the dendrite that carried the excitatory drive (Jadi et al. 2012; Pouille et al. 2013; Trevelyan and Watkinson 2005). These approaches would help, first, to validate the role of divisive gain modulation in entrainment, and second, to promote a more biophysical understanding of its effect.

Another limitation of this modeling study is that we did not explore how the constants of the input-output functions, θ*_e,i_* and α*_e,i_*, influence the effect of *q*. These constant values were taken directly from the original Wilson–Cowan equations as they were set for sustained oscillations (Wilson and Cowan 1972). Additionally, again as in the original study, there is only one time constant in our model. Arguably, different time constants for the different subpopulations would make the model more realistic and should be explored in future studies, especially a modeling study investigating the effect of different types of neuromodulators (fast GABA vs. slow GABA), for which different time constants have been used in the past (Taylor and Baier 2011; Wang et al. 2012), and their interaction with the type of the inhibition would be interesting. Furthermore, this study is limited to an input that targets only the excitatory (principal) population. In addition, the present model is limited to a regime of low-frequency oscillations. Future studies should investigate entirely different regimes (such as gamma-band oscillations), especially in the context of brain disorders, where gamma-band entrainment is of interest (see below). Finally, the self-inhibitory modulation of the *I*_soma_ subpopulation was modeled as subtractive, rather than divisive, mainly for simplicity. A divisive self-inhibition may have an impact on the oscillatory dynamics of the microcircuit, but, arguably, the most important interactions for generating and modulating oscillations are those between excitatory and inhibitory populations. Therefore, this study focused on the different modulations of the input-output function of the pyramidal neurons that, due to their distinct morphology, may allow for more diverse modulations (inhibition on the distal vs. proximal dendrites; Jadi et al. 2012; Pouille et al. 2013); see also inhibitory modulation from dendrite-targeting to soma-targeting interneurons (Cottam et al. 2013).

Interestingly, cellular adaptation works exactly the same way as subtractive inhibition: it shifts the input-output curve to higher values of input. However, synaptic depression, as modeled in Tabak et al. (2006, 2011), is implemented as an input gain modulation rather than an output gain modulation (as in our model). In both cases, the gain (slope) is dynamically changing, but only an output gain modulation decreases the maximal firing rate (Silver 2010; Womelsdorf et al. 2014). Another important difference between our model and the previous models is the interaction between the inhibitory components. In our model, the inhibitory populations directly interact to account for the interactions between inhibitory subpopulations in neocortical microcircuits (Pfeffer et al. 2013). Previous models lack these connections between inhibitory components (Tabak et al. 2006, 2011).

The effect of divisive gain modulation on entrainment, as seen in this theoretical study, suggests the importance of the soma-targeting interneurons and their modulatory effects in physiological brain dynamics. There is evidence linking subcellular alterations in soma-targeting interneurons with the phenotype of schizophrenia (for a review, see Nakazawa et al. 2012) and other neurological conditions, such as epilepsy (Rossignol et al. 2013; Tan et al. 2011). In fact, regarding the pathophysiology of epilepsy, there is evidence for the critical role of the inhibitory interneurons in both focal seizures (Eissa et al. 2018, 2017; Trevelyan and Schevon 2013) and in vitro models of epileptic discharges (Parrish et al. 2019; Trevelyan et al. 2006). Studies on brain tissue recovered from schizophrenia patients provide evidence of molecular alterations in soma-targeting interneurons resulting in lower expression of GAD67 and higher µ-opioid receptor expression, which leads to deficient synthesis and release of GABA (Curley and Lewis 2012; Lewis et al. 2012). Similar postmortem studies verified that these subpopulation abnormalities are mostly molecular and not structural (Glausier et al. 2014). These molecular alterations may impair divisive gain modulation and, in turn, entrainment, as observed in schizophrenia patients (Brenner et al. 2003; Hamm et al. 2015; Krishnan et al. 2009).

In conclusion, by using an extended version of the Wilson–Cowan model, we have shown that a gain control mechanism enhances the entrainment capabilities of neocortical microcircuits. The enhancement in entrainment reflects more flexible oscillatory dynamics, which is crucial for the adaptive functionality of the circuit (Schwab et al. 2006; Spiegler et al. 2011). We believe that the results in this study encourage further investigation of the role of gain modulation in entrainment through future computational and experimental studies.

## GRANTS

C. A. Papasavvas was supported by Wellcome Trust PhD Studentship 099755/Z/12/Z. Y. Wang gratefully acknowledges funding from Wellcome Trust Grants 208940/Z/17/Z and 210109/Z/18/Z. M. Kaiser and A. J. Trevelyan are supported by the CANDO project (http://www.cando.ac.uk/) funded through Wellcome Trust Grant 102037 and Engineering and Physical Sciences Research Council (EPSRC) Grant NS/A000026/1. M. Kaiser is additionally supported by Medical Research Council (MRC) Grant MR/T004347/1 and by the Portabolomics project funded through EPSRC Grant EP/N031962/1. A. J. Trevelyan is also supported by MRC Grant MR/R005427/1 and Biotechnology and Biological Sciences Research Council Grant BB/P019854/1.

## DISCLOSURES

No conflicts of interest, financial or otherwise, are declared by the authors.

## AUTHOR CONTRIBUTIONS

C.A.P. and Y.W. conceived and designed research; C.A.P. performed experiments; C.A.P. analyzed data; C.A.P., A.J.T., M.K., and Y.W. interpreted results of experiments; C.A.P. prepared figures; C.A.P. drafted manuscript; C.A.P., A.J.T., M.K., and Y.W. edited and revised manuscript; C.A.P., A.J.T., M.K., and Y.W. approved final version of manuscript.

## ENDNOTE

At the request of the authors, readers are herein alerted to the fact that additional materials related to this manuscript may be found at https://github.com/cpapasavvas/Entrainment2019. These materials are not a part of this manuscript and have not undergone peer review by the American Physiological Society (APS). APS and the journal editors take no responsibility for these materials, for the website address, or for any links to or from it.
